# Cyclic β^2,3^-amino acids improve the serum stability of macrocyclic peptide inhibitors targeting the SARS-CoV-2 main protease

**DOI:** 10.1093/bulcsj/uoae018

**Published:** 2024-03-06

**Authors:** Takashi Miura, Tika R Malla, Lennart Brewitz, Anthony Tumber, Eidarus Salah, Kang Ju Lee, Naohiro Terasaka, C David Owen, Claire Strain-Damerell, Petra Lukacik, Martin A Walsh, Akane Kawamura, Christopher J Schofield, Takayuki Katoh, Hiroaki Suga

**Affiliations:** Department of Chemistry, Graduate School of Science, The University of Tokyo, 7-3-1 Hongo, Bunkyo-ku, Tokyo 113-0033, Japan; Department of Chemistry and the Ineos Oxford Institute for Antimicrobial Research, Chemistry Research Laboratory, University of Oxford, 12 Mansfield Road, Oxford, OX1 3TA, United Kingdom; Department of Chemistry and the Ineos Oxford Institute for Antimicrobial Research, Chemistry Research Laboratory, University of Oxford, 12 Mansfield Road, Oxford, OX1 3TA, United Kingdom; Department of Chemistry and the Ineos Oxford Institute for Antimicrobial Research, Chemistry Research Laboratory, University of Oxford, 12 Mansfield Road, Oxford, OX1 3TA, United Kingdom; Department of Chemistry and the Ineos Oxford Institute for Antimicrobial Research, Chemistry Research Laboratory, University of Oxford, 12 Mansfield Road, Oxford, OX1 3TA, United Kingdom; Department of Chemistry, Graduate School of Science, The University of Tokyo, 7-3-1 Hongo, Bunkyo-ku, Tokyo 113-0033, Japan; Department of Chemistry, Graduate School of Science, The University of Tokyo, 7-3-1 Hongo, Bunkyo-ku, Tokyo 113-0033, Japan; Harwell Science & Innovation Campus, Diamond Light Source, Didcot, Oxfordshire, OX11 0DE, United Kingdom; Harwell Science & Innovation Campus, Research Complex at Harwell, Didcot, OX11 0FA, United Kingdom; Harwell Science & Innovation Campus, Diamond Light Source, Didcot, Oxfordshire, OX11 0DE, United Kingdom; Harwell Science & Innovation Campus, Research Complex at Harwell, Didcot, OX11 0FA, United Kingdom; Harwell Science & Innovation Campus, Diamond Light Source, Didcot, Oxfordshire, OX11 0DE, United Kingdom; Harwell Science & Innovation Campus, Research Complex at Harwell, Didcot, OX11 0FA, United Kingdom; Harwell Science & Innovation Campus, Diamond Light Source, Didcot, Oxfordshire, OX11 0DE, United Kingdom; Harwell Science & Innovation Campus, Research Complex at Harwell, Didcot, OX11 0FA, United Kingdom; Department of Chemistry and the Ineos Oxford Institute for Antimicrobial Research, Chemistry Research Laboratory, University of Oxford, 12 Mansfield Road, Oxford, OX1 3TA, United Kingdom; Chemistry—School of Natural and Environmental Sciences, Newcastle University, Newcastle upon Tyne, NE1 7RU, United Kingdom; Department of Chemistry and the Ineos Oxford Institute for Antimicrobial Research, Chemistry Research Laboratory, University of Oxford, 12 Mansfield Road, Oxford, OX1 3TA, United Kingdom; Department of Chemistry, Graduate School of Science, The University of Tokyo, 7-3-1 Hongo, Bunkyo-ku, Tokyo 113-0033, Japan; Department of Chemistry, Graduate School of Science, The University of Tokyo, 7-3-1 Hongo, Bunkyo-ku, Tokyo 113-0033, Japan

**Keywords:** β-amino acid, macrocyclic peptide, protease inhibition

## Abstract

Due to their constrained conformations, cyclic β^2,3^-amino acids (cβAA) are key building blocks that can fold peptides into compact and rigid structures, improving peptidase resistance and binding affinity to target proteins, due to their constrained conformations. Although the translation efficiency of cβAAs is generally low, our engineered tRNA, referred to as tRNA^Pro1E2^, enabled efficient incorporation of cβAAs into peptide libraries using the flexible in vitro translation (FIT) system. Here we report on the design and application of a macrocyclic peptide library incorporating 3 kinds of cβAAs: (1*R*,2*S*)-2-aminocyclopentane carboxylic acid (β^1^), (1*S*,2*S*)-2-aminocyclohexane carboxylic acid (β^2^), and (1*R*,2*R*)-2-aminocyclopentane carboxylic acid. This library was applied to an in vitro selection against the SARS-CoV-2 main protease (M^pro^). The resultant peptides, BM3 and BM7, bearing one β^2^ and two β^1^, exhibited potent inhibitory activities with IC_50_ values of 40 and 20 nM, respectively. BM3 and BM7 also showed remarkable serum stability with half-lives of 48 and >168 h, respectively. Notably, BM3A and BM7A, wherein the cβAAs were substituted with alanine, lost their inhibitory activities against M^pro^ and displayed substantially shorter serum half-lives. This observation underscores the significant contribution of cβAA to the activity and stability of peptides. Overall, our results highlight the potential of cβAA in generating potent and highly stable macrocyclic peptides with drug-like properties.

## Graphical Abstract

**Figure:**
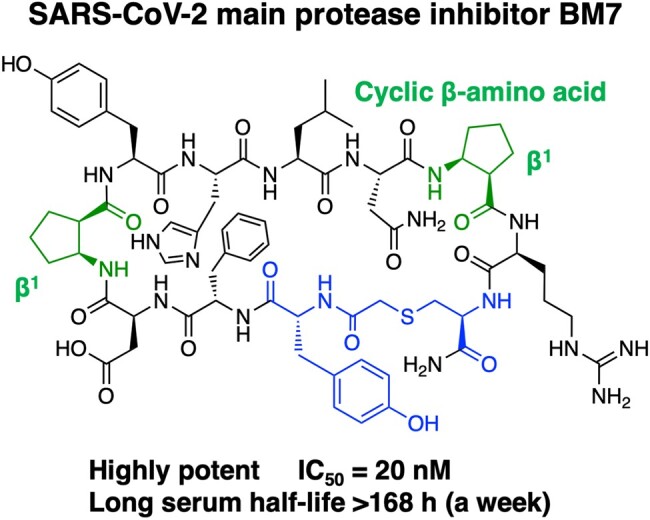
We report an in vitro selection of macrocyclic peptide inhibitors containing cyclic β^2,3^-amino acids (cβAA) against the SARS-CoV-2 main protease (M^pro^). The resulting peptides, including BM7 bearing 2 (1*R*,2*S*)-2-aminocyclopentane carboxylic acids (β^1^), exhibited highly potent inhibitory activities and remarkable serum stabilities due to the significant contribution of cβAAs.

## 1. Introduction

Natural translation machinery only utilizes the 20 proteinogenic α-amino acids (pAAs) as building blocks for protein (peptide) synthesis. However, genetic code reprogramming enables us to ribosomally synthesize peptides containing various nonproteinogenic amino acids (npAAs), including β-amino acids, in place of pAAs.^1,2^ To facilitate reprogramming, we have developed the flexible in vitro translation (FIT) system, which consists of precharged npAA-tRNAs prepared by using flexizymes and a reconstituted *Escherichia coli* translation system.^3,4^ Whereas the incorporation of various α-amino acid derivatives into nascent peptide chains has been established, the incorporation of β-amino acids has required considerable and prolonged efforts.^1,5–8^ In particular, the incorporation of multiple/consecutive β-amino acids has been extremely inefficient compared with that of a single β-amino acid.

**Fig. 1.**
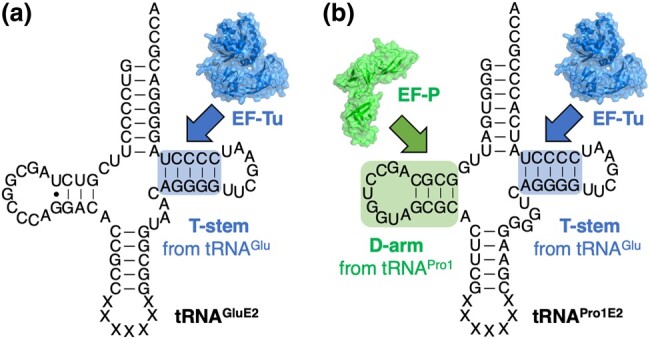
Secondary structures of tRNAs used for genetic code reprogramming. a) tRNA^GluE2^. b) tRNA^Pro1E2^. The T-stem motif for EF–Tu binding is in blue. The D-arm motif for EF–P binding is in green.

The difficulties of incorporating β-amino acids can be attributed to the following 2 reasons: (1) slow accommodation of npAA-tRNA onto the ribosomal A-site mediated by elongation factor thermo unstable (EF–Tu) and (2) slow peptidyl transfer of the P-site peptidyl-tRNA onto the A-site npAA-tRNA catalyzed by the peptidyl transferase center of the ribosome.^9^ The slow accommodation and slow peptidyl transfer can induce ribosomal stalling and mistranslocation of peptidyl-tRNA, resulting in peptidyl-tRNA drop-off. To accelerate the accommodation of npAA-tRNA (to address issue [1]), we engineered tRNA to be efficiently recognized by EF–Tu. Since the binding of aminoacyl-tRNA to EF–Tu is regulated by the T-stem region of tRNA, we designed an engineered tRNA, referred to as tRNA^GluE2^, in which the T-stem was replaced by that of *E. coli* tRNA^Glu^ with a high binding affinity for EF–Tu (Fig. 1a).^10^ The expression level of peptides containing β-amino acids was significantly improved using tRNA^GluE2^ compared with the use of weaker tRNAs.^11^ To promote peptidyl transfer between noncanonical residues (to address issue [2]), we introduced a proline-specific elongation factor, named EF–P, and optimized the D-arm region of tRNA. EF–P is known to promote peptidyl transfer reactions between consecutive prolines in nature.^12,13^ We have previously reported that EF–P recognizes Pro-tRNA^Pro^ by a specific D-arm motif of tRNA^Pro^ isoacceptors.^14^ We combined the T-stem and D-arm motifs into an engineered tRNA, namely tRNA^Pro1E2^ (Fig. 1b).^15^ Efficient incorporation of β-amino acids and their derivatives, including β^3^-amino acids, β^2,3^-amino acids, α-aminoxy acids, and α-hydrazino acids, has been accomplished using tRNA^Pro1E2^ in the presence of EF–P.^11,16,17^ Furthermore, we have successfully incorporated consecutive β-amino acids, such as up to 7 consecutive β-homomethionines and up to 10 consecutive (1*S*,2*S*)-2-aminocyclopentane carboxylic acids, which was not attainable with ordinary suppressor tRNAs, such as tRNA^AsnE2^.^7,18^ In addition to β-amino acids, various types of npAAs, such as D-amino acids, α,α-disubstituted amino acids, and γ-amino acids, have been efficiently incorporated using the devised translation system.^10,15,19,20^

**Fig. 2.**
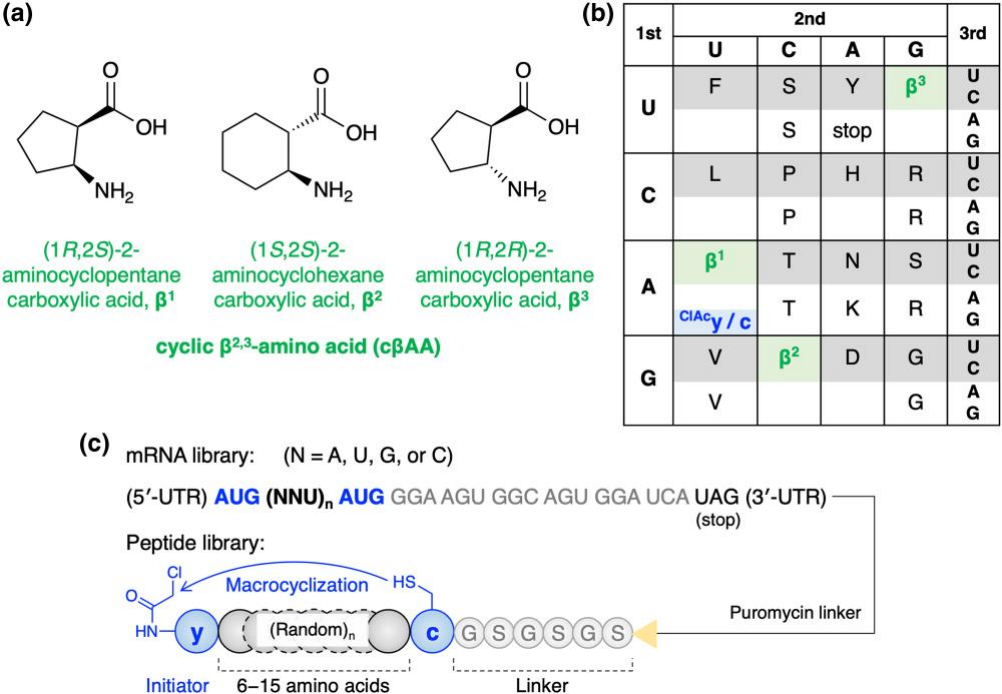
Incorporation of cyclic β-amino acids and D-amino acids into a peptide library by means of genetic code reprogramming. a) Structures of cβAAs used in this study. b) Reprogrammed codon table that consists of 3 cβAAs (β^1−3^), 2 D-α-amino acids (^ClAc^y and c), and pAAs. ^ClAc^y was assigned at the initiator AUG codon and the others were assigned at the elongator codons. c) Sequence design of mRNA library and the corresponding peptide library. Peptides spontaneously macrocyclized between ^ClAc^y and c via a thioether bond. The mRNA and peptide were covalently linked via a puromycin linker.

The FIT system can be applied to the ribosomal synthesis of random peptide libraries comprising over 10^12^ unique members with various β-amino acids. Such libraries are compatible with in vitro selection methodologies, such as mRNA display, for the discovery of bioactive peptides. In particular, the integration of the FIT system with mRNA display, referred to as the Random nonstandard Peptides Integrated Discovery (RaPID) system, enables us to efficiently screen potent peptide ligands containing npAAs.^5^ Our successful in vitro selection results have highlighted the contribution of npAAs, such as β-, γ-, D-α-, and *N*-methyl-α-amino acids, to improved bioactivity, proteolytic stability, and/or cell membrane permeability of peptides.^16,21–24^ Notably, cyclic β^2,3^-amino acids (cβAAs) (see Fig. 2a for the structures of representative cβAAs used in this study), which possess constrained cyclic structures, are interesting building blocks with defined folding propensities. cβAAs induce rigid secondary structures of peptides, such as 12-helix, 14-helix, 10/11/11-helix, 14/15-helix, β-turn, and γ-turn, referred to as foldamers, thereby improving proteolytic resistance and binding affinity to target proteins.^25–39^

Recently, we performed RaPID selections using macrocyclic peptide libraries containing cβAAs and cyclizing D-α-amino acids against 2 therapeutic targets: activated coagulation factor XII (FXIIa) and interferon-gamma receptor I (IFNGR1).^16,23^ The resulting highly potent inhibitors prompted us to further explore macrocyclic peptide inhibitors containing cβAAs against another important therapeutic target, SARS-CoV-2 main protease (M^pro^),^40,41^ also known as 3-chymotrypsin-like cysteine protease (3CL^pro^) and nonstructural protein 5 (nsp5), which plays an essential role in viral replication, cleaving viral polyproteins into functional proteins.^42^ Here we report studies aimed at obtaining macrocyclic peptides with high M^pro^ inhibitory activity and proteolytic stability from a ribosomally synthesized macrocyclic peptide library, taking advantage of the unique folding propensities of cβAAs that contribute to enhanced binding affinity and proteolytic stability.

## 2. Experimental

### 2.1 Preparation of tRNAs and flexizymes

tRNAs and flexizymes (dFx and eFx) were transcribed in vitro using the T7 RNA polymerase from the corresponding template DNAs prepared by using extension and the polymerase chain reaction (PCR) amplification (see Supporting Information [SI] Supplementary Table S1 for primer sequences). Extension and PCR were carried out using the following reaction mixture: 50 mM KCl, 10 mM Tris-HCl (pH 9.0), 0.1% (v/v) Triton X-100, 2.5 mM MgCl_2_, 250 μM dNTP mix, and 60 nM *Taq* DNA polymerase. 2 μM each of forward and reverse extension primers were added to the extension mixture and the reaction was initiated at 95 °C for 1 min followed by 5 cycles of 50 °C for 1 min and 72 °C for 1 min. The resulting solution and 0.5 μM each of forward and reverse PCR primers were added to the PCR mixture. PCR was performed for 15 cycles of 95 °C for 40 s, 55 °C for 40 s, and 72 °C for 40 s. The PCR product was purified by using phenol/chloroform extraction and ethanol precipitation. Transcription of RNA was carried out at 37 °C for 16 h using the following mixture: 40 mM Tris-HCl (pH 8.0), 22.5 mM MgCl_2_, 10 mM dithiothreitol, 1 mM spermidine, 0.01% Triton X-100, 3.75 mM (tRNAs) or 5 mM (flexizymes) nucleoside triphosphate (NTP) mix, 0.04 U/μL RNasin RNase inhibitor (Promega, N2615), and 120 nM T7 RNA polymerase. For transcription of tRNAs, 5 mM guanosine monophosphate (GMP) was added to the above solution to introduce a monophosphate at the 5′ end of the tRNAs. The resulting RNA transcripts were then treated with RQ1 DNase (Promega, M6101) at 37 °C for 30 min and purified by using 8% (tRNAs) or 12% (flexizymes) denaturing polyacrylamide gel electrophoresis (PAGE) containing 6 M urea.

### 2.2 Preparation of aminoacylated tRNAs by using flexizymes

cβAAs and D-cysteine (c) were pre-activated as their 3,5-dinitrobenzyl esters; *N*-chloroacetyl-D-tyrosine (^ClAc^y) was activated as its cyanomethyl ester.^4,16^ These activated amino acids were charged onto the respective tRNAs using flexizymes (dFx for 3,5-dinitrobenzyl ester or eFx for cyanomethyl ester). Aminoacylation was carried out at 4 °C for 6 h for c, for 16 h for cβAAs, or 2 h for ^ClAc^y in the following mixture: 600 mM MgCl_2_, 20% (v/v) dimethyl sulfoxide (DMSO), 25 μM dFx or eFx, 25 μM tRNA, and 5 mM activated amino acid. The reaction pH was adjusted by using bicine-KOH (pH 8.7) for cβAAs or HEPES-KOH (pH 7.5) for c and ^ClAc^y. The reaction was stopped by the addition of 4× volume of 0.3 M sodium acetate (pH 5.2) and 10× volume of ethanol. The resulting aminoacyl-tRNAs were subjected to ethanol precipitation by using centrifugation (15,000 × *g*, 25 °C, 15 min) and the pellets were washed with 70% ethanol.

### 2.3 Production of recombinant SARS-CoV-2 M^pro^

Recombinant M^pro^ was prepared as reported. Freeze/thawing cycles of M^pro^ were avoided so as not to compromise activity.^43,44^

### 2.4 RaPID selection of peptides against M^pro^

The random mRNA library was ligated with a puromycin linker at the 3′ end and added to the following translation mixture: 50 mM HEPES-KOH (pH 7.6), 100 mM KOAc, 12.3 mM Mg(OAc)_2_, 2 mM adenosine triphosphate (ATP), 2 mM guanosine triphosphate (GTP), 1 mM cytidine triphosphate (CTP), 1 mM uridine triphosphate (UTP), 20 mM creatine phosphate, 2 mM spermidine, 1 mM dithiothreitol, 1.5 mg/mL *E. coli* total tRNA, 1.2 μM *E. coli* ribosome, 2.7 μM initiation factor 1 (IF1), 3 μM IF2, 1.5 μM IF3, 20 μM elongation factor Tu/Ts (EF–Tu/Ts), 5 μM EF–P, 0.1 μM EF–G, 0.25 μM release factor 2 (RF2), 0.17 μM RF3, 0.5 μM ribosome recycling factor (RRF), 4 μg/mL creatine kinase, 0.1 μM T7 RNA polymerase, 3 μg/mL myokinase, 0.1 μM inorganic pyrophosphatase, 0.1 μM nucleotide diphosphate kinase, pAAs (500 μM each D, F, G, H, K, L, N, P, R, S, T, Y, and V), 0.03 μM ArgRS, 0.38 μM AsnRS, 0.13 μM AspRS, 0.09 μM GlyRS, 0.02 μM HisRS, 0.04 μM LeuRS, 0.11 μM LysRS, 0.68 μM PheRS, 0.16 μM ProRS, 0.04 μM SerRS, 0.09 μM ThrRS, 0.02 μM TyrRS, 0.02 μM ValRS, 20 μM c-tRNA^Pro1E2^_CAU_, 20 μM β^1^-tRNA^Pro1E2^_GAU_, 20 μM β^2^-tRNA^Pro1E2^_GGC_, 20 μM β^3^-tRNA^GluE2^_GCA_, and 20 μM ^ClAc^y-tRNA^fMet^_CAU_ (SI Supplementary Fig. S1A, Step 1). The peptide library was translated at 37 °C for 40 min in 150 μL (for the first round of selection) or 5 μL (from the second round) of the FIT system and incubated at 25 °C for 5 min to conjugate the translated peptide with the corresponding mRNA/puromycin (Step 2). 0.04× volume of 500 mM EDTA (pH 8.0) was then added and incubated at 37 °C for 10 min to dissociate ribosomes from the mRNA/peptide conjugates. Reverse transcription (42 °C, 30 min) used the PCR reverse primer (see SI Supplementary Table S1 for the sequence) and M-MLV reverse transcriptase lacking RNase H activity (Promega, M3682; Step 3). The resulting cDNA/mRNA/peptide conjugates were subjected to naked Dynabeads M-280 Streptavidin (Thermo Fisher, DB11206) treatment (4 °C, 15 min) 3 times to remove bead-binding peptides; the supernatant was then applied to M^pro^-immobilized Dynabeads (4 °C, 15 min; Step 4). The beads were washed with 100 μL ice-cold TBS-T buffer (50 mM Tris-HCl (pH 7.6), 150 mM NaCl, 0.05% (v/v) Tween 20) 3 times. Note that the removal of bead-binding peptides was not performed for the first selection round. 100 μL of 1× PCR buffer (10 mM Tris-HCl [pH 9.0], 50 mM KCl, 0.1% [v/v] Triton X-100, 0.25 mM dNTP, 2.5 mM MgCl_2_, 0.25 μM each PCR forward and reverse primers) was added to the beads; the cDNAs were eluted at 95 °C for 5 min and PCR amplified to make a cDNA library (Step 5). To estimate the recovery rate of cDNA, 1 μL of the elute was mixed with 19 μL of 1× PCR buffer containing SYBR Green I (Lonza, 50513) and *Taq* DNA polymerase; amounts of cDNA were quantified by using real-time PCR.

### 2.5 Solid-phase peptide synthesis

Macrocyclic peptides were chemically synthesized on a milligram scale via standard Fmoc solid-phase peptide synthesis using a Syro I automated peptide synthesizer (Biotage). NovaPEG Rink Amide Resin (54 mg, 25 μmol) was incubated with *N,N*-dimethylformamide (DMF) at room temperature for 1 h. Each Fmoc-protected amino acid was coupled at 30 °C for 40 min on the resin in a DMF solution containing 0.2 M Fmoc-protected amino acid (6 equiv.), 0.2 M 2-(1*H*-benzotriazole-1-yl)-1,1,3,3-tetramethyluronium hexafluorophosphate (HBTU; 5 equiv.), 0.2 M 1-hydroxybenzotriazole (HOBt; 5 equiv.), and 0.1 M *N,N*-diisopropylethylamine (DIPEA; 12 equiv.). After the resin was washed 5 times with 600 μL DMF, the Fmoc group was deprotected with 600 μL of 40% (v/v) piperidine in DMF at 30 °C for 12 min. Coupling of the Fmoc-protected amino acid and Fmoc deprotection were repeated as required. After automated peptide synthesis, 0.2 M chloroacetyl *N*-hydroxysuccinimide ester (8 equiv.) in *N*-methylpyrrolidone was added to the resin; the mixture was incubated at room temperature for 1 h with rotation. After the resin was subsequently washed with DMF 3 times and with dichloromethane 5 times, the resin-bound peptides were treated with 2 mL of a solution of 92.5% (v/v) trifluoroacetic acid (TFA), 2.5% (v/v) water, 2.5% triisopropylsilane (TIS), and 2.5% 3,6-dioxa-1,8-octanedithiol (DODT) at room temperature for 3 h with rotation to deprotect the side-chain protecting groups and to cleave the peptide off from the resin. The resulting linear peptides were precipitated with diethyl ether, then dissolved in 10 mL of 80% (v/v) DMSO, 20% (v/v) water, and 0.1% (v/v) TFA. Following the addition of 200 μL of 0.5 M tris(2-carboxyethyl) phosphine (TCEP) and triethylamine to adjust the pH to 8, the peptide mixture was incubated with rotation at room temperature for 16 h to form a thioether bond between the N-terminal chloroacetamide and the thiol group of the downstream cysteine. Macrocyclization of the peptides was confirmed by using matrix-assisted laser desorption/ionization time-of-flight mass spectrometry (MALDI-TOF MS); the crude peptides were purified by using reverse-phase high performance liquid chromatography (HPLC) (Shimadzu) with a Chromolith Prep RP-18 column (Merck).

### 2.6 Evaluation of binding affinity of peptides by using surface plasmon resonance

The binding affinities of peptides to M^pro^ were evaluated through surface plasmon resonance (SPR) using a Biacore T200 instrument (Cytiva) at 25 °C with the following running buffer: 10 mM Tris-HCl (pH 8.0), 150 mM NaCl, 0.05% (v/v) Tween 20, and 0.1% (v/v) DMSO. Biotin-tagged M^pro^ was immobilized on a sensor chip CAP (Cytiva) to a surface density of 1,000 to 1,500 response units following the immobilization protocols provided by Cytiva. The kinetic constant was determined using a single-cycle kinetics method by the injection of 5 different concentrations (2-fold dilution series) of each peptide at a flow rate of 30 μL/min. The resulting sensorgram was fitted to the standard 1:1 interaction model and analyzed using the Biacore evaluation software (Cytiva).

### 2.7 Solid-phase extraction coupled to MS inhibition assays

Inhibition of M^pro^ was measured by solid-phase extraction (SPE) purification coupled to MS analysis using a RapidFire (RF) 365 high-throughput sampling robot (Agilent) connected to an iFunnel Agilent 6550 accurate mass quadrupole time-of-flight (Q-TOF) spectrometer as reported.^43,45^ In brief, the cyclic peptides were dispensed in an 11-point, 3-fold dilution series (top concentration of 25 to 5 μM) using an acoustic Echo Dispenser machine (LabCyte). Formic acid and DMSO were used as positive and negative inhibition controls, respectively. The assays were performed using isolated recombinant SARS-CoV-2 M^pro^ (75 nM), which was prepared as reported,^43,44^ and a 37-mer peptide substrate (ALNDFSNSGSDVLYQPPQTSITSAVLQ/SGFRKMAFPS-NH_2_; 4 µM). Reactions were incubated (15 min), then quenched by the addition of 10% (v/v) aqueous formic acid (5 μL/well).

### 2.8 Serum stability assays

A synthetic macrocyclic peptide (10 μM) and a serum-resistant internal standard peptide consisting only of D-amino acids (NH_2_-PEG_5_-wstndwstnd-PEG_5_-CONH_2_, 5 μM) were mixed and incubated in human serum (Cosmo Bio, 12181201) at 37 °C for up to 168 h.^16^ At each time point, 4 μL of the mixture was removed and quenched by adding 12 μL of methanol, followed by incubation on ice for 15 min. Following centrifugation (15,000 × *g*, 25 °C, 10 min), 10 μL of the supernatant was mixed with 40 μL of 1% (v/v) TFA in water. Following centrifugation (15,000 × *g*, 25 °C, 5 min), the supernatant was analyzed using LC/MS employing a reverse-phase column (ACQUITY UPLC BEH C18, 1.7 μm, 2.1 × 150 mm; Waters) and a Xevo G2-XS QTof system (Waters) with a linear gradient from 1% B to 61% B. Buffer A: water with 0.1% (v/v) formic acid; buffer B: acetonitrile with 0.1% (v/v) formic acid. The percentages of remaining peptides were determined by using the peak area integration of the chromatograms. The obtained LC/MS data were analyzed using a MassLynx 4.1 (Waters).

## 3. Results and discussion

### 3.1 RaPID selection of macrocyclic peptide binders to M^pro^

Taking advantage of tRNA^GluE2^ and tRNA^Pro1E2^, we constructed a macrocyclic peptide library containing 3 kinds of cβAAs: (1*R*,2*S*)-2-aminocyclopentane carboxylic acid (β^1^), (1*S*,2*S*)-2-aminocyclohexane carboxylic acid (β^2^), and (1*R*,2*R*)-2-aminocyclopentane carboxylic acid (β^3^) (Fig. 2). β^1^ and β^2^ were assigned to AUU and GCU codons using tRNA^Pro1E2^_GAU_ and tRNA^Pro1E2^_GGC_, respectively. β^3^ was introduced at the UGU codon using tRNA^GluE2^_GCA_, because EF–P inhibits translation of β^3^ when using tRNA^Pro1E2^.^16^ For macrocyclization of the peptides, *N*-chloroacetyl-D-tyrosine (^ClAc^y) and D-cysteine (c) were introduced at the initiator and elongator AUG codons using tRNA^fMet^_CAU_ and tRNA^Pro1E2^_CAU_, respectively. The thiol group of the c residue spontaneously reacts with the N-terminal chloroacetyl group of ^ClAc^y to form a thioether bond for macrocyclization. Each npAA was precharged onto the respective tRNA using flexizymes. The peptide library comprised a repeat of 6 to 15 random residues encoded by using NNU codons (N = A, U, G, or C) flanked by the cyclizing ^ClAc^y and c residues, followed by a GSGSGS linker connected to the 3′ end of the mRNA via a puromycin linker (Fig. 2c). The 3 cβAAs and the 12 pAAs (D, F, G, H, L, N, P, R, S, T, Y, and V) were assigned to the NNU codons.

The macrocyclic peptide library was then applied to the RaPID selection against recombinant SARS-CoV-2 M^pro^. The random mRNA library was translated into the peptide library followed by conjugation of the peptide with the parent mRNA via a puromycin linker (SI Supplementary Fig. S1A). The mRNA/peptide conjugates were reverse transcribed into mRNA/cDNA/peptide complexes and applied to the affinity selection. The library was first subjected to naked magnetic beads to remove bead-binders and then applied to biotin-tagged M^pro^ immobilized on streptavidin magnetic beads to recover M^pro^-binders. The bound fractions were recovered and amplified into the cDNA library by using PCR, followed by transcription into the mRNA library for the next selection round. By repeating the affinity selection, the recovery rate of M^pro^-binders increased at the third round, whereas that of bead-binders did not increase (SI Supplementary Fig. S1B).

**Fig. 3.**
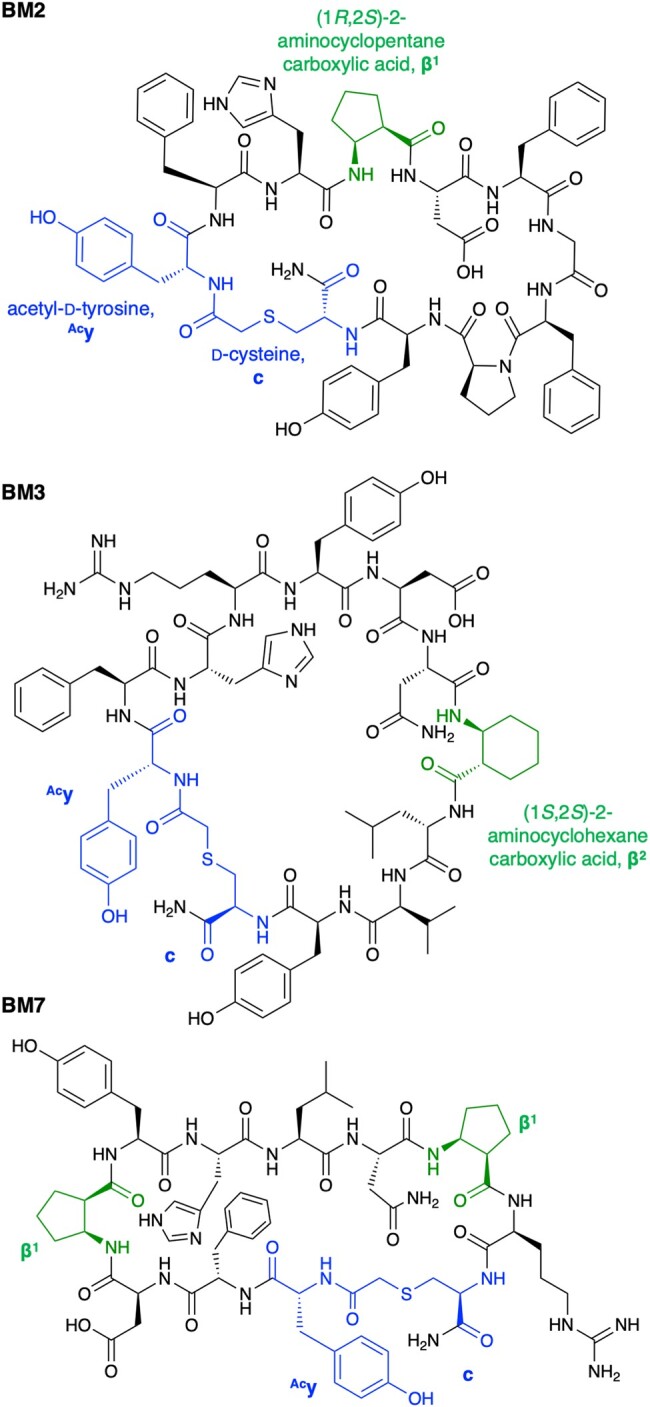
Structure of representative M^pro^ inhibitors BM2, BM3, and BM7.

The cDNA sequences and the corresponding peptides after the 3 rounds of selection were analyzed using next-generation sequencing (SI Supplementary Table S2 shows the top 100 sequences). Thirteen peptides containing β^1^ or β^2^ in their sequences were among the top 100 peptides, whereas β^3^ was not found in the major family. Of the 13 peptides, we chose 4 peptides containing the yFHβ^1^ motif at their N-termini (BM1, BM2, BM5, and BM6), 2 peptides containing a single β^1^ and β^2^ without the yFHβ^1^ motif (BM4 and BM3, respectively), and 1 peptide containing 2 β^1^ (BM7) for further analysis of their binding affinity, inhibitory activity, and serum stability (Table 1). BM1−7 were chemically synthesized using the standard solid-phase method without the C-terminal GSGSGS linker and their identities were confirmed by using MALDI-TOF MS (Fig. 3, SI Supplementary Figs. S2 and S3).

**Table 1.**
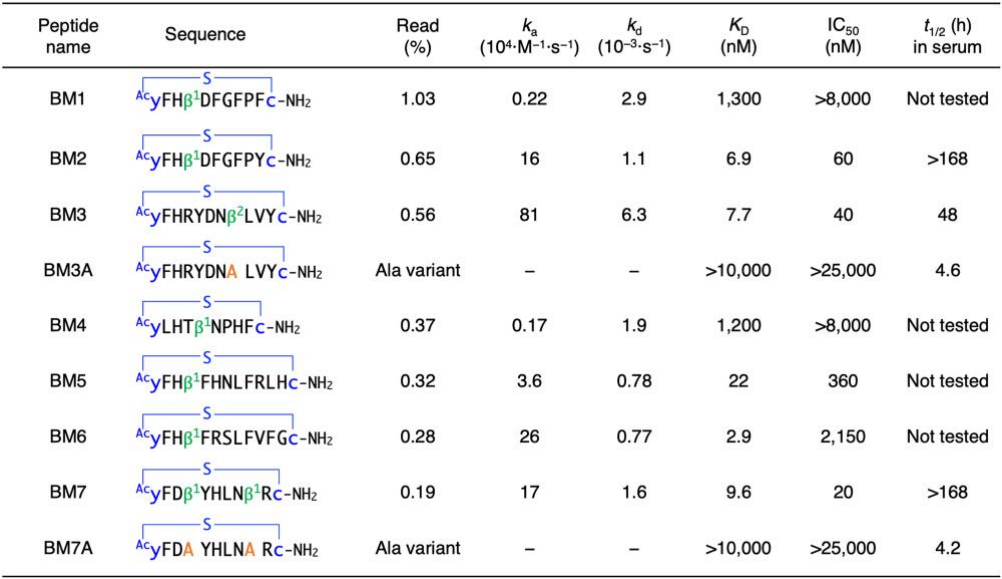
Binding affinity, inhibitory activity, and serum stability of peptide inhibitors and their variants. The thioether bond is shown as a blue line. See Fig. 3 and SI Supplementary Fig. S3 for the structures of peptides. Sequences, read (%), kinetic association (*k*_a_), dissociation (*k*_d_), equilibrium (*K*_D_), half-maximal inhibitory concentration (IC_50_), and half-life in human serum (*t*_1/2_) are shown. −: the kinetic values could not be accurately determined due to low affinity.

### 3.2 Biochemical activities and stabilities of peptide inhibitors

**Fig. 4.**
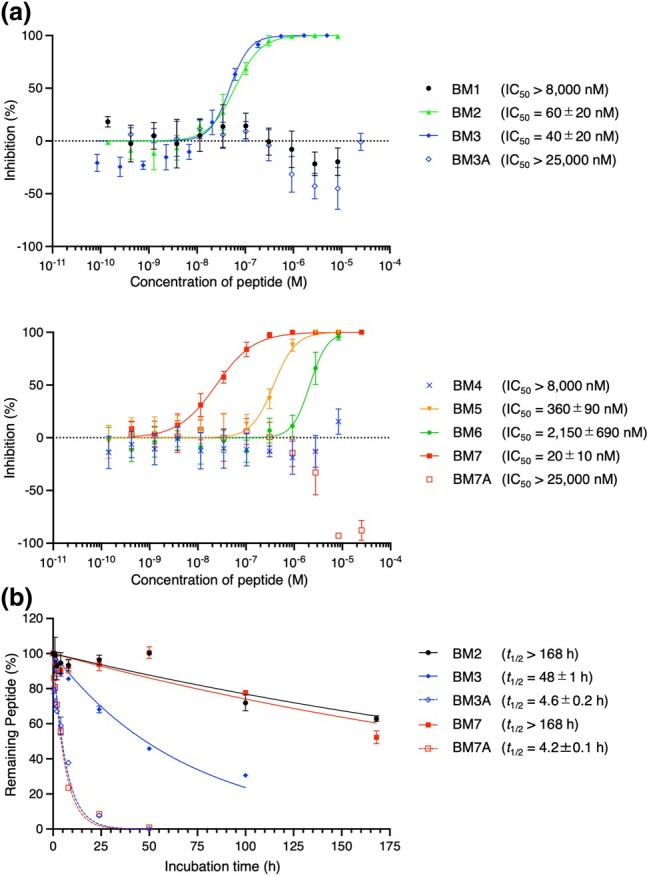
Inhibitory activity and serum stability of BM1−7 and their variants. a) Dose response analysis of peptides against M^pro^. The M^pro^ inhibitory activities of peptides were investigated by using SPE purification coupled to MS analysis. Data are presented as mean values ± standard deviation, s.d. (*n* = 3−5). b) Serum stability assay of macrocyclic peptides. Each peptide and an internal standard peptide were co-incubated in human serum at 37 °C and the relative amount of each peptide to standard was estimated by using LC/MS analysis at each time point. Data are presented as mean values ± s.d. (*n* = 3).

We first evaluated the binding affinities of BM1−7 to M^pro^ by using SPR. BM2, BM3, BM5, BM6, and BM7 exhibited strong binding affinities with *K*_D_ values of 2.9 to 22 nM (Table 1 and SI Supplementary Fig. S4). Surprisingly, BM1 and BM4 showed substantially lower binding affinity with *K*_D_ values of 1,300 and 1,200 nM, respectively, despite having high read frequencies. We next evaluated the inhibitory activity of BM1−7 against the hydrolytic activity of SARS-CoV-2 M^pro^ using a reported MS-based method (Fig. 4a).^43^ BM2, BM3, and BM7, which have single-digit nanomolar *K*_D_ values, showed particularly potent inhibition, with IC_50_ values of 60, 40, and 20 nM, respectively. Peptides with weaker binding affinity exhibited IC_50_ of 360 nM for BM5 and no inhibition for BM1 and BM4, implying a correlation between IC_50_ and *K*_D_ values. BM6 is an exception where the IC_50_ value of 2,150 nM was not consistent with the *K*_D_ value of 2.9 nM, likely due to its binding to a noncompetitive site of substrate. Notably, substituting the C-terminal tyrosine residue of BM2 for a phenylalanine completely abolished both binding and inhibition, highlighting the importance of single amino acid residues/specific functional groups for potent inhibition (Table 1); it is likely that the tyrosine hydroxyl group is involved in protein binding.

To evaluate contributions of the cβAA residues to potency, we synthesized variants of potent inhibitors with the cβAA being substituted for alanine (BM3A and BM7A, Table 1). Notably, the binding affinity and inhibitory activity of the alanine variants were completely lost, indicating that cβAA residues, namely β^2^ in BM3 and β^1^ in BM7, are essential for tight binding to M^pro^.

We also evaluated the half-life of potent cβAA-containing peptides and their variants in human serum, because the in vivo stability of peptide is one of the major issues in the development of peptide therapeutics.^46^ In general, peptides consisting only of L-α-amino acids are rapidly degraded in the range of minutes to a few hours by proteases in vivo.^47^ Each peptide and an uncleavable internal standard peptide were co-incubated in human serum at 37 °C; the relative amount of the remaining sample peptide was estimated by using LC/MS. The potent inhibitors BM2, BM3, and BM7, with IC_50_ values of 20 to 60 nM, exhibited remarkably high peptidase resistance with half-lives (*t*_1/2_) of more than 168 h (equivalent to a week), 48 h, and more than 168 h, respectively (Table 1, Fig. 4b, and SI Supplementary Fig. S5). By contrast, the alanine variants BM3A and BM7A showed substantially lower stabilities with half-lives of 4.6 and 4.2 h, respectively, highlighting the essential roles of cβAAs not only for efficient binding and inhibitory activity, but also for enhancing the proteolytic stability of peptides, probably because their folding ability stabilizes peptide structures. Our previous reports suggested that macrocyclic peptides are primarily digested by in-serum trypsin-like proteases at the C-terminal to Arg residues.^24,48^ BM7 has an Arg residue flanked by nonproteinogenic β^1^ and c, which likely prevent the recognition of the Arg residue by proteases, resulting in its remarkably long serum half-life (more than 168 h). On the other hand, BM3 has a shorter half-life of 48 h compared with BM7, likely due to the protease susceptibility of the Arg residue distant away from β^2^ by 4 amino acid residues. Unlike BM7 and BM3, BM2 exhibited a longer serum half-life (more than 168 h), whose resistance can be attributed to the absence of Arg in the sequence.

## 4. Conclusion

Our results highlight the significant contributions of β^1^ and β^2^ residues in the highly potent M^pro^ inhibitory peptides BM7 and BM3 to their remarkable inhibitory activities (IC_50_ of 20 and 40 nM, respectively) and serum stabilities (*t*_1/2_ of more than 168 and 48 h, respectively). As shown in our previous crystallographic study of a FXIIa inhibitor, F3, in which 2 β^2^ residues contributed to its inhibitory activity and stability via the formation of β- and γ-turn structures,^16^ the strong folding abilities of β^1^ and β^2^ found in BM7 and BM3 are also expected to be crucial for their potency. The ribosomally mediated synthesis of libraries containing such nonstandard peptides, with multiple cβAAs and D-amino acids, was previously considered near impossible. However, the breakthrough development of tRNA^Pro1E2^ has enabled us to prepare a custom-made FIT system including EF–P that has allowed the preparation of such “foldamer-type” libraries, opening up an entirely new chemical space relative to that obtainable by using the natural transcription/translation machinery.

Compared with our macrocyclic peptides BM3 and BM7, other M^pro^ inhibitory peptides reported to date exhibit limited activity and/or stability. A substrate-derived cyclic peptide inhibitor (UCI-1^49^) and linear L-peptide inhibitors (p13^50^ and compound 21^51^) have been reported; however, their inhibitory activities are in the micromolar range. Eberle et al. designed all-D-peptide inhibitors based on the retro-inverso principle from phage display hit L-peptides, resulting in metabolically stable D-peptides with moderate inhibitory activity (IC_50_ up to 1.57 μM).^52,53^ Johansen-Leete et al. screened potent thioether macrocyclic peptides from a ribosomally synthesized library consisting of standard pAAs in the random region and cyclizing ^ClAc^y and L-Cys by means of the RaPID system (IC_50_ up to 70 nM).^54^ Despite their high potency, stability against hydrolysis by M^pro^ is limited, with 30% degradation after 1 h of incubation with 2.5 μM M^pro^, highlighting the importance of our results for the design of hydrolysis stable inhibitors of M^pro^ and other disease-relevant enzymes. Xu et al. discovered potent disulfide macrocyclic L-peptides from a virtual screening (IC_50_ up to 19 nM), although the lability of the disulfide bond under the physiological reducing conditions would eliminate their active forms and their serum stability has not yet been defined but is likely poor.^55^

The reported results indicate the difficulty of achieving high inhibitory activity against proteases and proteolytic resistance with peptides consisting only of α-amino acids. On the other hand, cβAAs are excellent building blocks that stabilize peptide structures with a minimal number of residues. In fact, although BM3 and BM7 have only 1 β^2^ and 2 β^1^, respectively, they exhibited 2-digit nanomolar IC_50_ values and remarkably long serum half-lives. We recently reported potent macrocyclic peptide M^pro^ inhibitors containing cyclic γ^2,4^-amino acids (cγAA) that have rigid cyclic main chains.^24^ One resultant peptide, GM4, comprising 13 residues with a cγAA, exhibited an IC_50_ value of 50 nM and a serum half-life of 126 h. A crystal structure of the M^pro^:GM4 complex revealed that the noncanonical cγAA residue binds to the catalytic subsite as a small amino acid surrogate and prevents hydrolysis by M^pro^. This observation underscores the potential of cγAAs as another promising building block of peptide therapeutics. Therefore, the combined use of cβAA and cγAA within a single peptide is a possible future strategy for discovering novel macrocyclic peptides characterized by both high potency and exceptional stability. These peptides hold great potential for diverse therapeutic applications, representing a captivating frontier in peptide-based drug development.

## Supplementary data

Supplementary material is available at *Bulletin of the Chemical Society of Japan* online.

## Supplementary Material

uoae018_Supplementary_Data

## Notes

**Takashi Miura**

Takashi Miura is a PhD student at the University of Tokyo working under the supervision of Professor Hiroaki Suga. He has been awarded the JSPS Research Fellowship for Young Scientists (DC2), World-leading Innovative Graduate Study Program for Co-designing Future Society (WINGS-CFS), and Designing Future Society (DFS) Fellowship. His research focuses on the in vitro selection of macrocyclic peptide inhibitors containing β- and γ-amino acids.

## Notes

**Hiroaki Suga**

Hiroaki Suga received PhD degree from Massachusetts Institute of Technology in 1994. After postdoctoral work in Massachusetts General Hospital, he started his independent academic career in the Department of Chemistry of the State University of New York, University at Buffalo from 1997 to 2003. He was then a professor at the Research Center for Advanced Science and Technology in the University of Tokyo. He received his current position at the same institute in 2010. His work focuses on the development of biochemical platform technologies related to peptides, proteins, and therapeutic applications.
