# Supplementary material for: Cyclic β2,3-amino acids improve the serum stability of macrocyclic peptide inhibitors targeting the SARS-CoV-2 main protease
Source: Bull Chem Soc Jpn. 2024 Mar 6;97(5):uoae018. doi: 10.1093/bulcsj/uoae018 (PMC11141402; doi:10.1093/bulcsj/uoae018)
Supplement: uoae018_Supplementary_Data [file uoae018_supplementary_data.zip › bcsj-2023-0313-File004.pdf]

Supporting Information for:

Cyclic β[2,3]-amino acids improve the serum stability of macrocyclic peptide inhibitors targeting the SARS-CoV-2 main protease

Takashi Miura,[1] Tika R. Malla,[2] Lennart Brewitz,[2] Anthony Tumber,[2] Eidarus Salah,[2] Kang Ju Lee,[1] Naohiro Terasaka,[1] C. David Owen,[3,4] Claire Strain-Damerell,[3,4] Petra Lukacik,[3,4] Martin A. Walsh,[3,4] Akane Kawamura,[2,5] Christopher J. Schofield,[2] Takayuki Katoh,[1] and Hiroaki Suga*[1]

[1]Department of Chemistry, Graduate School of Science, The University of Tokyo, 7-3-1 Hongo, Bunkyo-ku, Tokyo 113-0033, Japan

[2]Department of Chemistry and the Ineos Oxford Institute for Antimicrobial Research, Chemistry Research Laboratory, University of Oxford, 12 Mansfield Road, OX1 3TA, Oxford, United Kingdom

[3]Diamond Light Source, Harwell Science & Innovation Campus, Didcot, Oxfordshire OX11 0DE, United Kingdom

[4]Research Complex at Harwell, Harwell Science & Innovation Campus, Didcot OX11 0FA, United Kingdom

[5]Chemistry – School of Natural and Environmental Sciences, Newcastle University, Newcastle upon Tyne, NE1 7RU, United Kingdom.

E-mail: hsuga@chem.s.u-tokyo.ac.jp

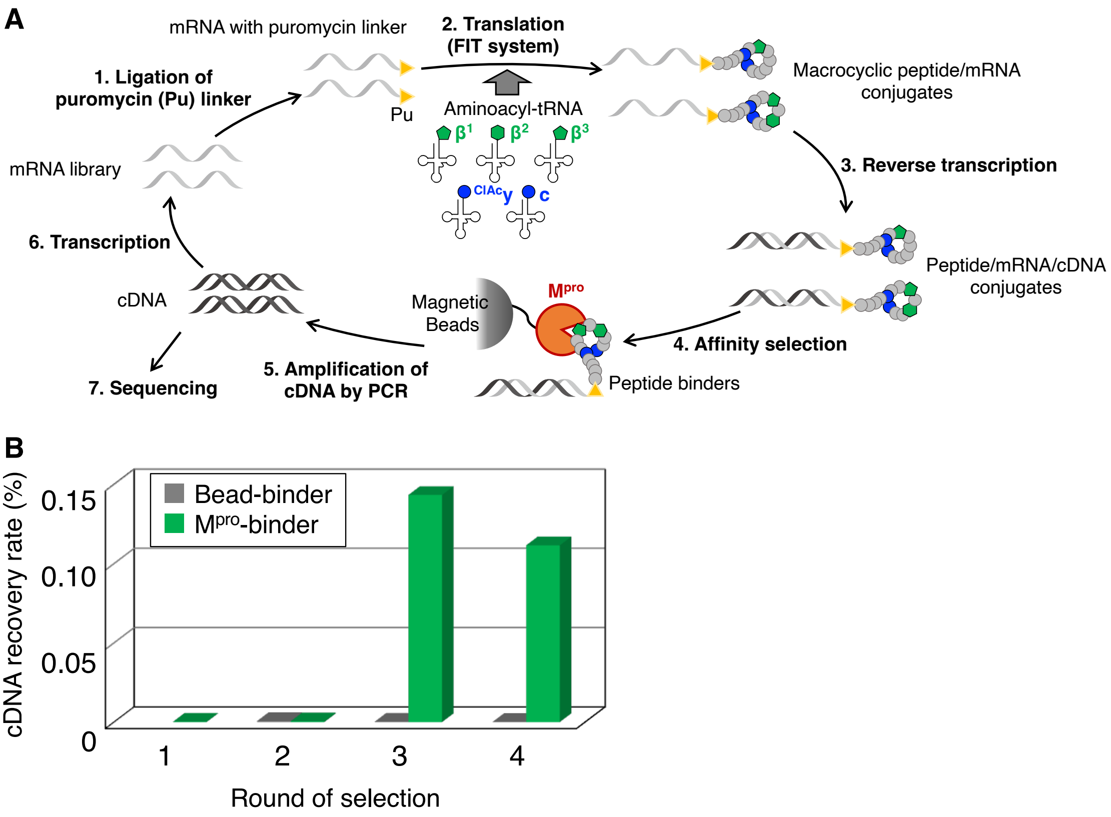

Figure S1. RaPID selection against IFNGR1 using ribosomally synthesized macrocyclic peptide libraries. (A) Schematic depiction of RaPID selection. 1) Ligation of a puromycin linker to the 3′-end of the mRNA library. 2) Translation of peptides using the reprogrammed genetic code with nonproteinogenic aminoacyl-tRNAs, followed by macrocyclization of peptide between ^{ClAc}y and c via a thioether bond. 3) Reverse transcription of mRNA into cDNA. 4) Affinity selection of peptides against naked magnetic beads, followed by selection against IFNGR1 immobilized beads. 5) Recovery of the bound fraction and amplification of cDNA of binders by PCR. 6) Transcription of cDNA into mRNA. 7) Next generation sequencing analysis of cDNA. (B) Recovery rate of cDNA after the affinity selection at each round. Green and gray bars indicate the recovery rate of M^{pro}-binders and bead-binders, respectively. Bead-binder selection was not performed in the first round.

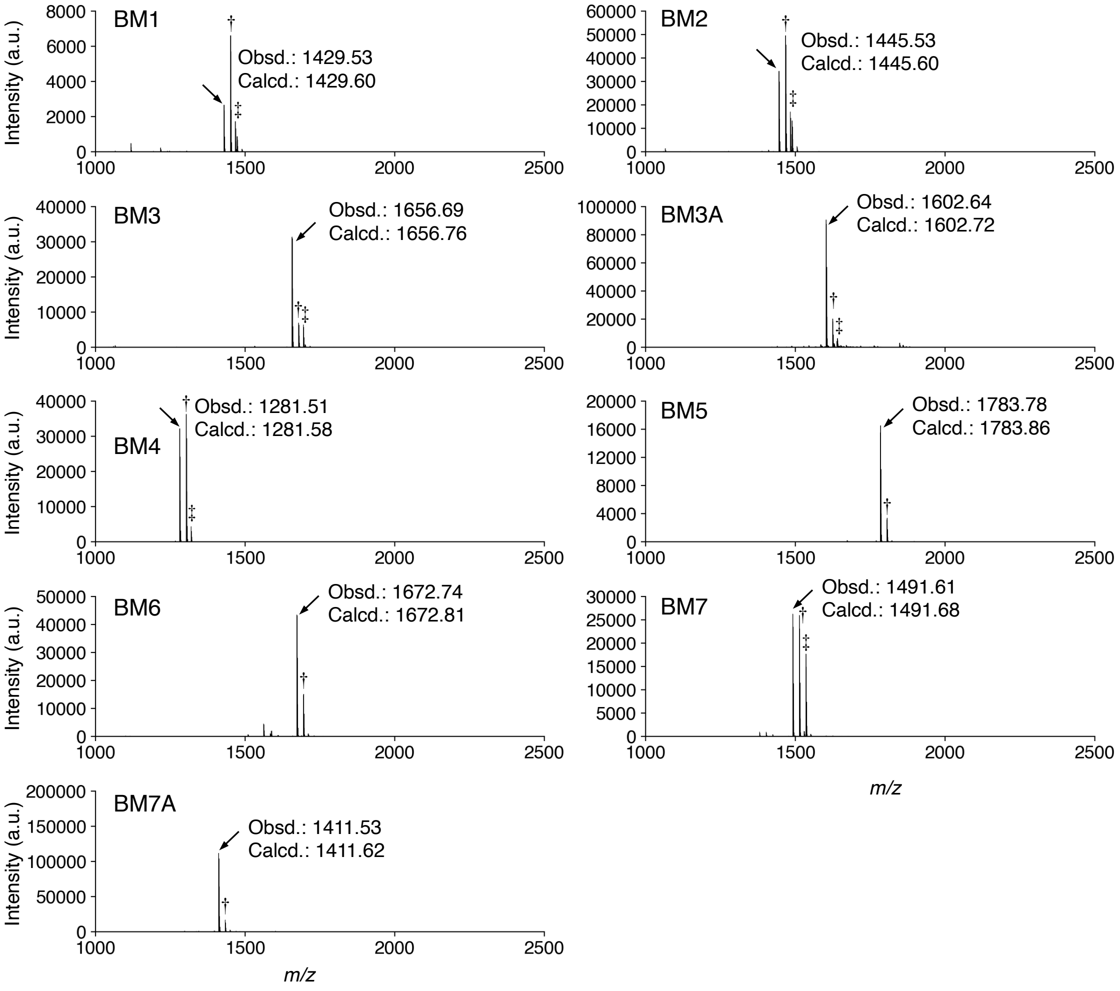

Figure S2. Identities of chemically synthesized peptides. MALDI-TOF mass spectra of peptides. Calcd. and Obsd. indicate calculated and observed $[M+H]^+$ values, respectively. † and ‡ indicate sodium and potassium ion adducts, respectively.

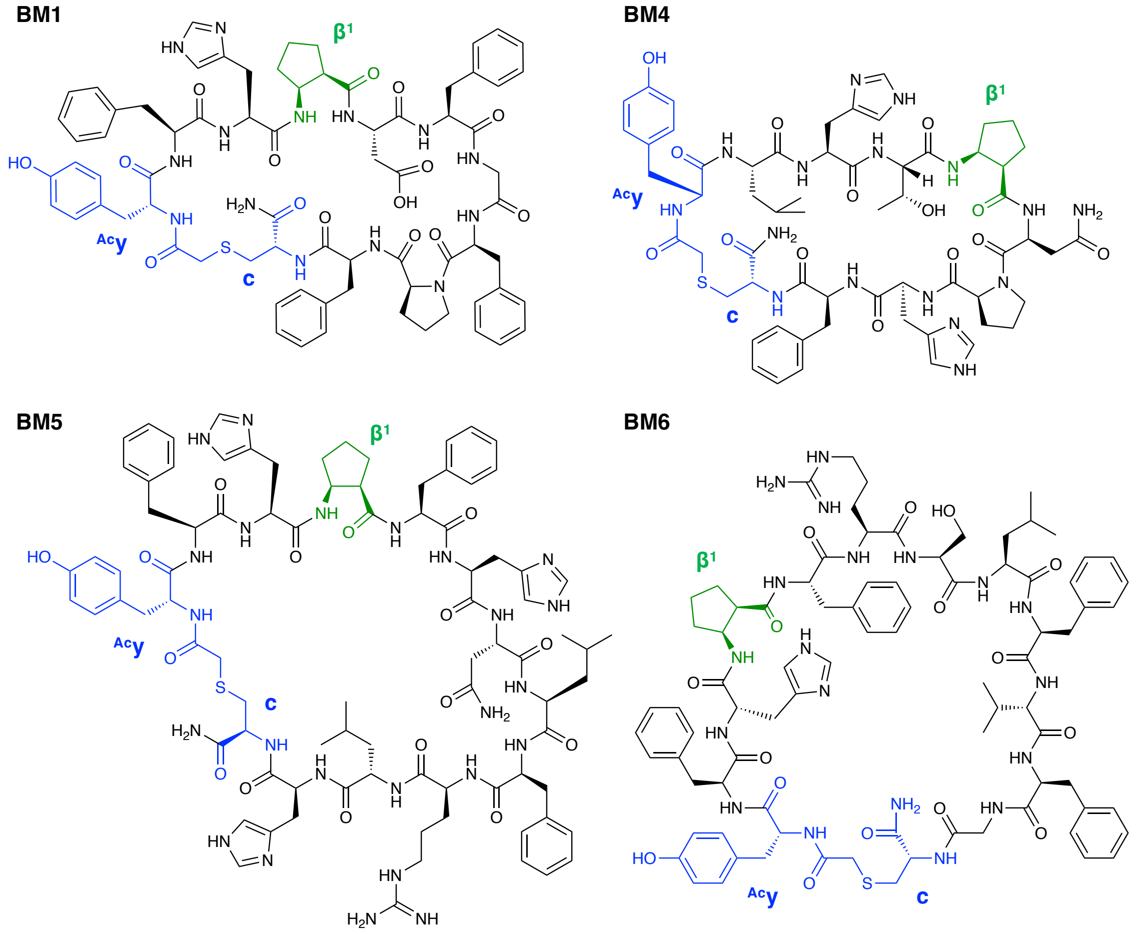

Figure S3. Structures of selected macrocyclic peptides. See Figure 3 for BM2, BM3 and BM7 structures.

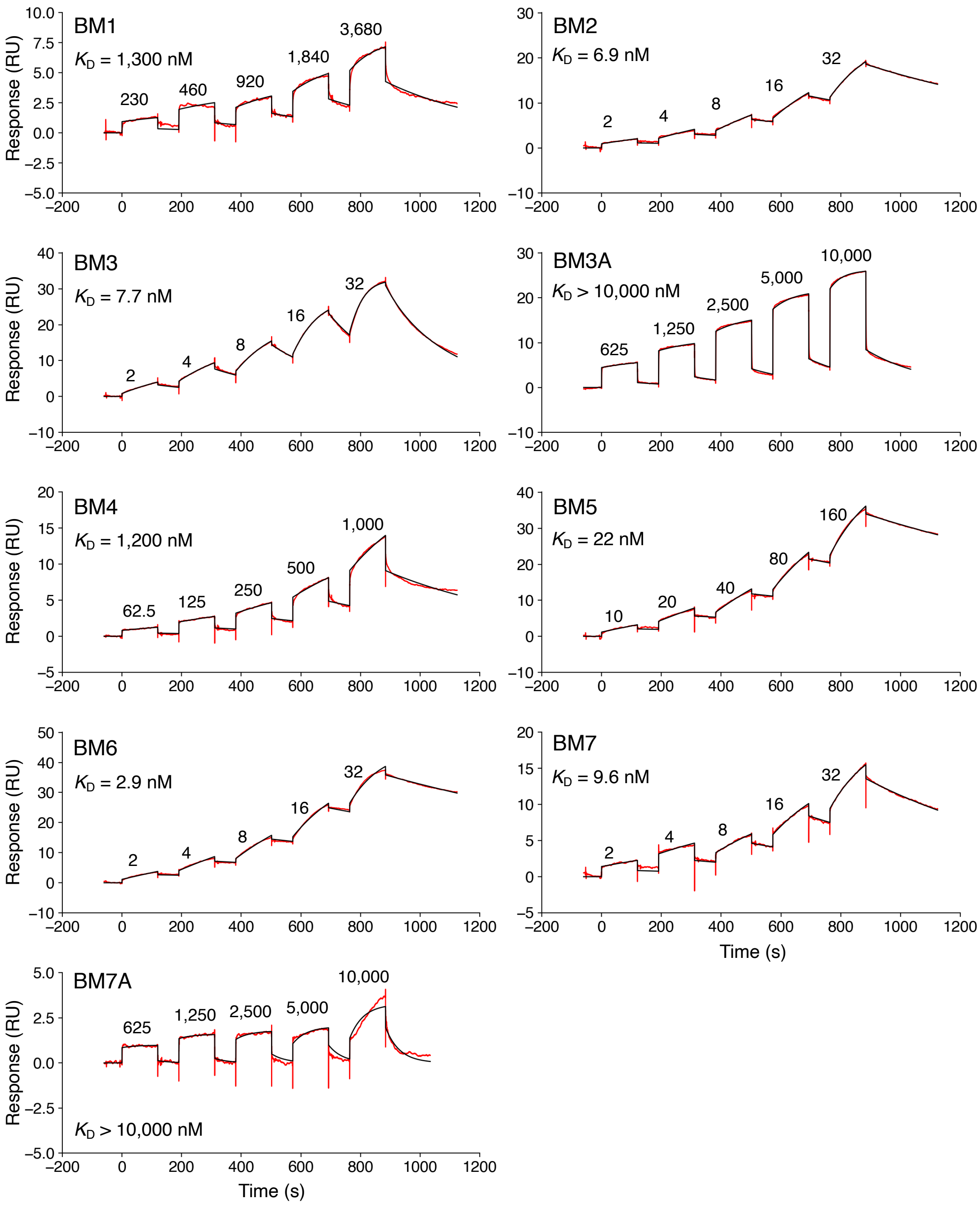

Figure S4. Binding kinetics of macrocyclic peptides against M^{pro}. SPR sensorgrams of selected peptides and variants. The sequences and kinetic values are shown in Table 1. Five different concentrations (nM), indicated by the numbers above the sensorgrams, of each peptide were injected for measuring kinetic constants. Binding sensorgrams were fitted using the standard 1:1 binding model. Red and black lines indicate raw sensorgrams and fitted curves, respectively.

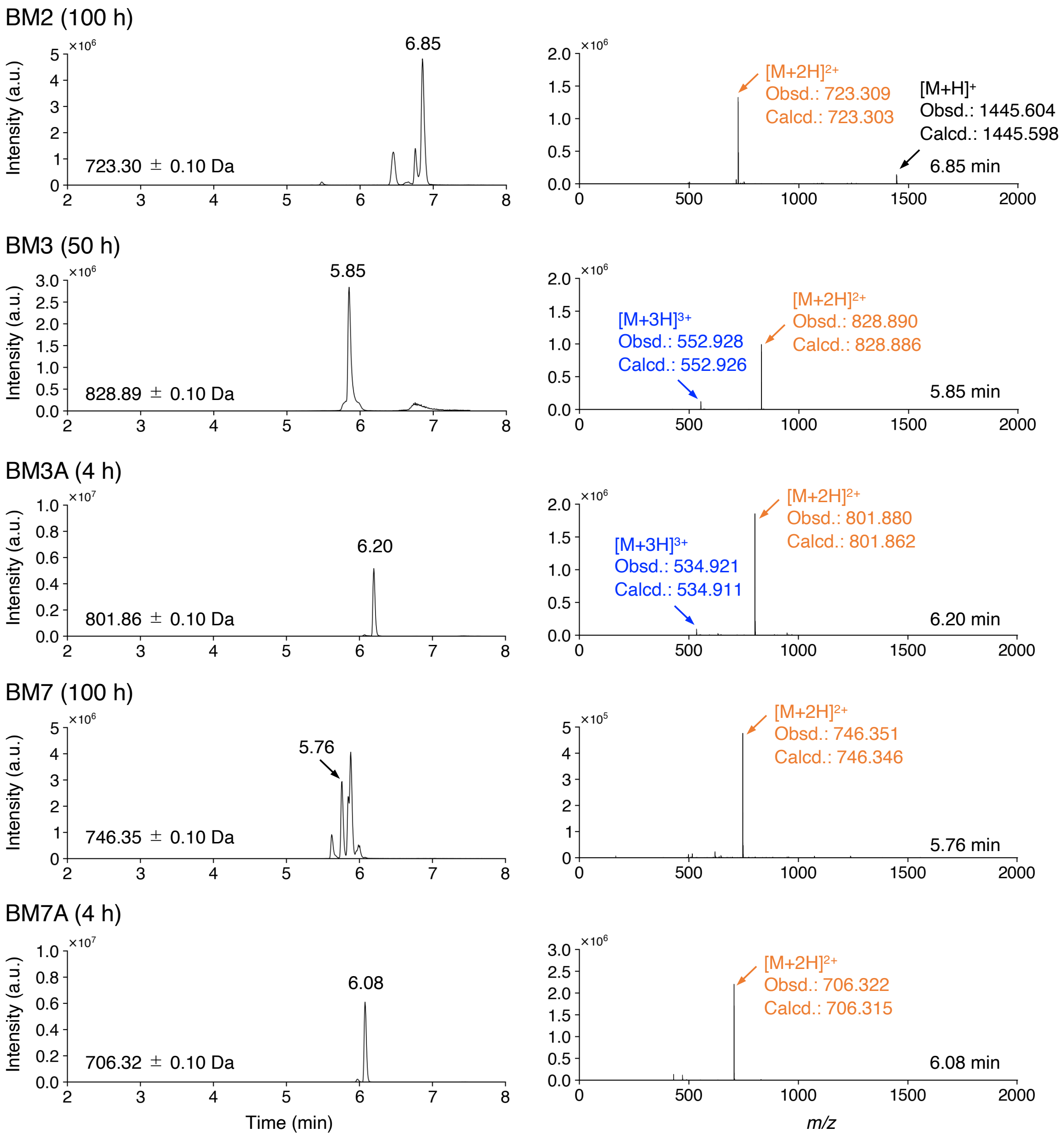

Figure S5. Serum stability assay of macrocyclic peptides. Each peptide and an internal standard peptide were co-incubated in human serum at 37 °C, and relative amount of each peptide to standard peptide was estimated by LC/MS at each time point. Extracted-ion chromatogram and mass spectra of reaction mixture of serum stability assay are shown. Black, orange, and blue arrows indicate $[M+H]^{+}$, $[M+2H]^{2+}$, and $[M+3H]^{3+}$ ions, respectively. Calcd. and Obsd. indicate calculated and observed *m/z* values, respectively.
